# 

**Published:** 1857-10

**Authors:** 


*A word to our Subscribers.*

In the July No., the seventh of the present volume, we sent bills to all who had not paid their subscriptions to the end of this year. Many responded promptly by sending their money, and to such we feel under renewed obligations. But, two more numbers will bring the year to a close, and there are many on our subscription list who still remain indebted not only for the present volume, but many of them for one, two, or three preceding volumes also. The number of our subscribers is abundantly sufficient, if they would pay promptly, to defray all the current expenses of publishing the *Journal*, and enable us to employ such help as would very much improve its contents. Hence it is as much for their interest as ours, to pay promptly for each current volume during the year of its publication.

We wish every delinquent subscriber would take the hint, and at once remit us by mail the amount due. If letters containing money are properly *registered*, we will cheerfully bear all the risk of losses by transmission.

### RECEIPTS ON SUBSCRIPTIONS,

UP TO OCT. 10TH, 1857.

**ILLINOIS.**

| | | |
|---|---|---|
| Dr. N. G. Stevens, | vol. 6, - | $2.00 |
| " C. N. Irwin - | " 5&6 - | 4.00 |
| " E. P. Cook - | " 6, - | 2.00 |
| " Bailey Ragon, | " " - | 2.00 |
| " W. R. Griswold, | " " - | 2.00 |
| " T. A. Hill, - - | " 5&6 - | 4.00 |
| " W. Leachman, | " 6&7 - | 5.00 |
| " Levi Propst, - | " 7, - | 2.00 |
| " P. Dickenson - | " 5&6 - | 4.00 |
| " S. H. Whitlesey, | " 6 - | 2.00 |

**MISSOURI.**

| | | |
|---|---|---|
| Dr. C. W. Snow, | vol. 5&6 - | $4.00 |
| " J. M. Starnes, | - - - - | 5.00 |

**INDIANA.**

| | | |
|---|---|---|
| Dr. R. B. Denny | vol. 6, - | $2.00 |
| " C. Angel, - | " 4,5&6, | 6.00 |
| " W. Townsend, | " 6, - | 2.00 |
| " J. E. Walker, - | " " - | 2.00 |

**MICHIGAN.**

| | | |
|---|---|---|
| Dr. S. H. Bristol, | vol. 5&6, | $4.00 |

**IOWA.**

| | | |
|---|---|---|
| Dr. W. A. Morse, | vol. 6, - | $2.00 |

**LOUISIANA.**

| | | |
|---|---|---|
| Dr. G. J. Huey, - | vol. 6, - | $2.00 |

**PENNSYLVANIA.**

| | | |
|---|---|---|
| Dr. John R. Finley, | vol. 5&6 | $5.00 |

